# The latest version ChatGPT powered by GPT-4o: what will it bring to the medical field?

**DOI:** 10.1097/JS9.0000000000001754

**Published:** 2024-06-10

**Authors:** Nan Zhang, Zaijie Sun, Yuchen Xie, Haiyang Wu, Cheng Li

**Affiliations:** a Department of Gynecological Tumor Ward, The Third Affiliated Hospital of Zhengzhou University; b Department of Orthopaedics, The First Affiliated Hospital of Zhengzhou University, Zhengzhou; c Xiangya Medical College, Central South University, Changsha, Hunan; d Department of Orthopaedics, Xiangyang Central Hospital, Affiliated Hospital of Hubei University of Arts and Science, Xiangyang; e Department of Clinical College of Neurology, Neurosurgery and Neurorehabilitation, Tianjin Medical University, Tianjin; f Department of Spine Surgery, Wangjing Hospital, China Academy of Chinese Medical Sciences, Beijing, China; g Center for Musculoskeletal Surgery (CMSC), Charité-Universitätsmedizin Berlin, Corporate Member of Freie Universität Berlin, Humboldt University of Berlin, Berlin Institute of Health, Berlin, Germany

*Dear Editor,*

To the best of our knowledge, *International Journal of Surgery* has published many studies on the potential applications of ChatGPT in multiple medical fields so far^1,2^. ChatGPT, a progeny of InstructGPT, descends from the GPT-3.5 lineage and has been meticulously refined via human-curated responses elicited by prompts within the OpenAI application programming interface (API) playground. The emergence of ChatGPT is lauded by numerous experts as a transformative innovation, heralding the commencement of a new epoch in artificial intelligence (AI). On 14 March 2023, OpenAI announced the release of a significantly improved version, GPT-4. Compared to GPT-3.5, the most notable feature of this new version lies in its enhanced ability to process images and text, which may fundamentally alter the way humans interact with computers^3^. Many clinical trials currently known are also tested based on GPT-4^3–5^. It is worth noting that this is not the end of the ChatGPT story. On 14 May 2024, OpenAI launched a new generation flagship generation model, GPT-4o. The letter ‘o’ stands for omni, a step toward more natural human–computer interaction. Once the news was released, it quickly caused a sensation around the world.

Compared with previous versions, GPT-4o has the following advantages and potential applications in the medical field: Firstly, GPT-4o exhibits performance in English text and code processing comparable to that of GPT-4 Turbo (a more advanced version of GPT-4 with a larger context window), yet it demonstrates substantial improvements in handling non-English texts. Additionally, the API operates at increased speed and reduces costs by 50%. This advancement signifies that, whether in crafting intricate code or processing diverse English texts, GPT-4o delivers performance on par with the most advanced models available. Concurrently, its enhanced capabilities in processing non-English texts render it more effective and versatile in a global multilingual environment. For instance, during multinational academic medical conferences requiring real-time translation and dialogue, GPT-4o significantly enhances communication efficacy among international researchers.

Beyond language processing advancements, the optimization of GPT-4o’s API speed is another significant highlight. With technological progress, user demands for faster response times have escalated. GPT-4o’s API operates significantly faster than its predecessors, enabling users to receive responses more swiftly upon model invocation. This enhancement not only enriches user experience but also expands its application scenarios. In particular, applications necessitating real-time data processing and rapid content generation greatly benefit from GPT-4o’s high-speed responsiveness. For example, GPT-4o may be able to integrate patients’ health data, including electronic health records, wearable device data, etc., conduct comprehensive analysis, and provide personalized health management recommendations.

In terms of visual and auditory comprehension, GPT-4o excels remarkably. Traditional AI models often face challenges in processing multimodal data, such as coordinating and understanding different data types. GPT-4o, however, showcases exceptional capability in this domain. It adeptly handles not only single data types but also efficiently processes combinations of text, audio, and images, subsequently generating corresponding outputs. This multimodal processing prowess allows GPT-4o to be applied in more intricate and diversified scenarios. For example, in the medical field, doctors could input patient symptoms via voice, and combined with the patient’s imaging data, GPT-4o is able to generate detailed diagnostic reports and treatment recommendations. This comprehensive data processing capacity ensures GPT-4o transcends single-task execution, integrating multiple information sources to provide patients with more accurate and holistic services.

Moreover, GPT-4o demonstrates remarkable efficiency in response times. It can respond to audio inputs in as little as 232 ms, with an average response time of 320 ms, closely mirroring human conversational response times. This near-instantaneous responsiveness renders GPT-4o exceptionally effective in applications requiring rapid reactions. In contexts such as voice assistants and real-time translation, prompt response times are crucial for user satisfaction. GPT-4o’s swift responsiveness ensures minimal perceptible delay during user interaction, significantly enhancing the overall user experience. Prior to the release of GPT-4o, users engaging with ChatGPT’s voice dialogue functionality experienced an average delay of 2.8 s with GPT-3.5 and 5.4 s with GPT-4. Comparatively, GPT-4o’s substantially reduced response times not only elevates system utility but also unlock new possibilities for real-time applications. In telemedicine, for instance, the quality of real-time dialogue between doctors and patients is paramount. GPT-4o’s rapid response capability facilitates smoother and more efficient communication in remote medical consultations.

Overall, GPT-4o excels not only in language and code processing but also in multimodal data processing, responsiveness, and cost-effectiveness. Its emergence marks another major leap in AI technology, providing stronger support and wider possibilities for various medical application scenarios. Both patients and doctors could benefit from GPT-4o’s technological advancements and experience more efficient and smarter medical care. With the widespread application of GPT-4o, we could expect AI to bring more innovation and changes in various fields of medical care.

## Ethical approval

This study does not include any individual-level data and thus does not require any ethical approval.

## Source of funding

This study is supported by China Postdoctoral Science Foundation (2022M720385) and Beijing JST Research Funding (YGQ-202313).

## Author contribution

N.Z.: conceptualization, methodology, data curation, formal analysis, resources, investigation, and writing – original draft; Z.S. and Y.X.: conceptualization, methodology, formal analysis, and investigation; H.W. and C.L.: conceptualization, methodology, formal analysis, resources, investigation, and writing – review and editing.

## Conflicts of interest disclosure

The authors declare no conflicts of interest.

## Research registration unique identifying number (UIN)

1. Name of the registry: not applicable.
2. Unique identifying number or registration ID: not applicable.
3. Hyperlink to your specific registration (must be publicly accessible and will be checked): not applicable.

## Guarantor

Nan Zhang, Haiyang Wu, and Cheng Li.

## Data availability statement

The data underlying this article will be shared by the corresponding author on reasonable request.
